# Inhibition of the cGAS‐STING pathway ameliorates the premature senescence hallmarks of Ataxia‐Telangiectasia brain organoids

**DOI:** 10.1111/acel.13468

**Published:** 2021-08-30

**Authors:** Julio Aguado, Harman K. Chaggar, Cecilia Gómez‐Inclán, Mohammed R. Shaker, Hannah C. Leeson, Alan Mackay‐Sim, Ernst J. Wolvetang

**Affiliations:** ^1^ Australian Institute for Bioengineering and Nanotechnology The University of Queensland Saint Lucia Queensland Australia; ^2^ Department of Neurogenetics Kolling Institute Sydney Medical School University of Sydney Sydney New South Wales Australia; ^3^ Griffith Institute for Drug Discovery Griffith University Nathan Queensland Australia; ^4^ Present address: Cellesce Ltd, Cardiff Medicentre Heath Park Cardiff UK

**Keywords:** Ataxia‐Telangiectasia, brain aging, brain organoids, cGAS‐STING signalling, cellular senescence, neurodegeneration

## Abstract

Ataxia‐telangiectasia (A‐T) is a genetic disorder caused by the lack of functional ATM kinase. A‐T is characterized by chronic inflammation, neurodegeneration and premature ageing features that are associated with increased genome instability, nuclear shape alterations, micronuclei accumulation, neuronal defects and premature entry into cellular senescence. The causal relationship between the detrimental inflammatory signature and the neurological deficiencies of A‐T remains elusive. Here, we utilize human pluripotent stem cell‐derived cortical brain organoids to study A‐T neuropathology. Mechanistically, we show that the cGAS‐STING pathway is required for the recognition of micronuclei and induction of a senescence‐associated secretory phenotype (SASP) in A‐T olfactory neurosphere‐derived cells and brain organoids. We further demonstrate that cGAS and STING inhibition effectively suppresses self‐DNA‐triggered SASP expression in A‐T brain organoids, inhibits astrocyte senescence and neurodegeneration, and ameliorates A‐T brain organoid neuropathology. Our study thus reveals that increased cGAS and STING activity is an important contributor to chronic inflammation and premature senescence in the central nervous system of A‐T and constitutes a novel therapeutic target for treating neuropathology in A‐T patients.

## Graphical abstract

Aguado et al. show that senescent astrocytes upregulate detrimental pro‐inflammatory SASP factors in a cGAS/STING‐dependant manner that promote accelerated ageing in brain organoids of ataxia‐telangiectasia. Pharmacological interventions in these organoids with cGAS and STING inhibitors reduce astrocyte senescence and the SASP, and consequently, improve neuronal activity and survival.

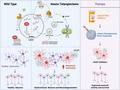

## 1 INTRODUCTION

Ataxia‐telangiectasia (A‐T) is a rare human genetic disease caused by homozygous or compound heterozygous mutations in the ataxia‐telangiectasia‐mutated (*ATM*) gene that encodes the ATM kinase. In agreement with ATM’s well‐documented role in orchestrating the cellular response to DNA double‐strand breaks (DSBs) (Shiloh & Ziv, 2013) A‐T patient cells show markedly increased radiosensitivity (Taylor et al., 2019), increased genome instability, elevated inflammation profiles, accelerated telomere attrition, mitochondrial dysfunction and premature entry into cellular senescence as compared to their healthy control counterparts (Shiloh & Lederman, 2017).

Uncontrolled or sustained inflammation is strongly linked to a number of age‐related chronic neurodegenerative diseases (Baker & Petersen, 2018), and a systemic elevation of inflammatory cytokine levels was previously linked to detrimental outcomes in both A‐T patients and mouse models. Interestingly, senescent cells adopt a pro‐inflammatory secretome known as the senescence‐associated secretory phenotype (SASP) (Campisi & d'Adda di Fagagna, 2007), which is proposed to play an important role in promoting neurological deficiencies (Bussian et al., 2018).

As cells enter into senescence, a major hallmark of human ageing (Lopez‐Otin et al., 2013), DNA fragments leak out from the nucleus into the cytoplasm of mammalian cells (Dou et al., 2017). Such cytoplasmic chromatin fragments—often seen in the form of micronuclei—are proposed to arise as a result of replication stress (Ho et al., 2016), DNA damage (Ahn et al., 2014), mitochondrial dysfunction (Vizioli et al., 2020) or intermediates of reverse‐transcribed retroelements (De Cecco et al., 2019). This results in activation of the cGAS‐STING pathway (Dou et al., 2017), a cytosolic DNA sensing signalling response that plays essential roles in activating pro‐inflammatory genes (Barber, 2015). Interestingly, A‐T patient skin fibroblasts prematurely undergo cellular senescence and accumulate micronuclei in culture (Lan et al., 2019). In agreement with these data, a recent report postulated a role for cGAS and STING in sensing cytoplasmic DNA in Atm^−/−^ mice, resulting in a systemic inflammatory phenotype (Hartlova et al., 2015) reminiscent of the clinical picture of A‐T patients.

Neurodegeneration remains however an important yet poorly understood aspect of A‐T (Rothblum‐Oviatt et al., 2016). Given that Atm^−/−^ mice generally fail to display clear symptoms of neurological deficiencies including neurodegeneration (Lavin, 2013), understanding how a lack of ATM kinase activity impacts the nervous system in humans is instrumental for understanding and treating A‐T‐associated neurodegeneration. In an effort to more accurately model the neurological detrimental phenotypes of A‐T, in this study, we used A‐T patient olfactory neurosphere‐derived (ONS) cells (Stewart et al., 2013) and generated induced pluripotent stem cells (iPSC) from these cells in order to generate human cortical brain organoids. Here, we show that primary A‐T patient ONS cells retain misshapen nuclei and exhibit significantly increased amounts of cytoplasmic DNA in the form of micronuclei, which in turn prime a constitutive SASP response that is dependent on the cGAS‐STING pathway. Furthermore, we then demonstrate that A‐T patient iPSC‐derived brain organoids show a similar increase in micronuclei, elevated numbers of senescent cells located mostly within astrocyte populations, increased expression of pro‐inflammatory genes, as well as premature neuronal degeneration and dysfunction. Importantly, inhibition of cGAS and STING‐dependent inflammation effectively suppresses self‐DNA‐induced SASP activation and rescues neuropathological features in A‐T brain organoids. Collectively, our results reveal a major contribution of the cGAS‐STING innate immune signalling pathway to detrimental neurological phenotypes of A‐T and validate its functional inhibition as a promising target for treating A‐T neuropathogenesis.

## 2 RESULTS

### 2.1 Micronuclei in primary A‐T ONS cells activate the cGAS‐STING pathway

**FIGURE 1:**
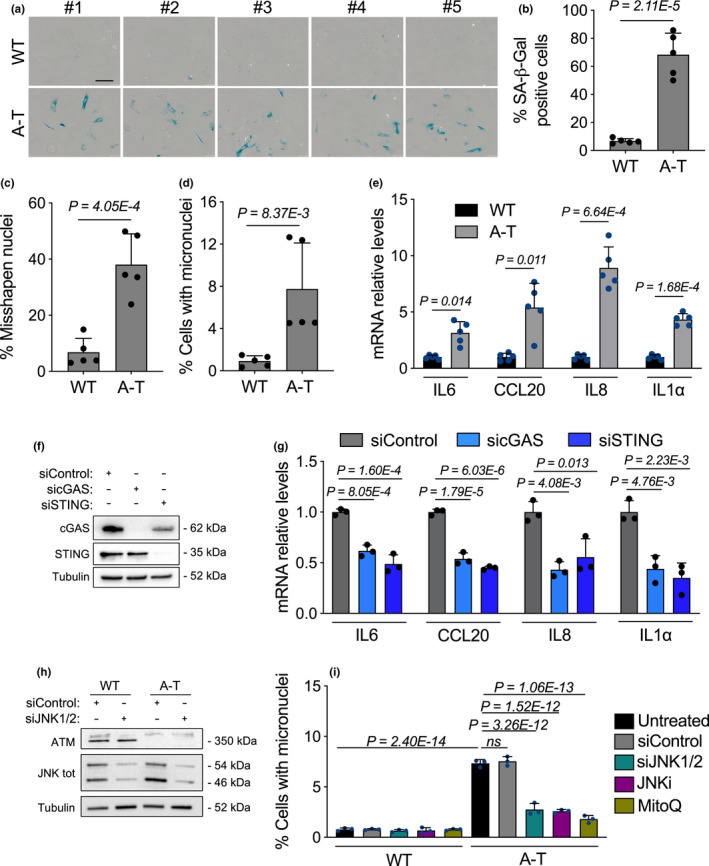
Lack of functional ATM in ONS patient cells induces a cGAS‐STING‐dependent SASP response driven by accumulation of cytoplasmic DNA. (a‐e) 5 wild type (WT) and 5 A‐T human ONS cell lines were used and analysed at identical passage number (passage 20). (a) Representative images of senescence‐associated β‐galactosidase (SA‐β‐gal) positive assays shown in b. Scale bar, 100 μm. (b–d) Each point in the bar plot in (b) shows the average percentage of SA‐β‐gal positive cells of one cell line, (c) the average percentage of cells with misshapen nuclei and in (d) the average percentage of cells with micronuclei. Error bars represent SD; *n* = 5 independent patient samples; Student's *t* test. (e) Total RNA from human A‐T ONS patient cells was used to quantify the mRNA expression levels of the indicated SASP genes and normalized to RPLP0 mRNA and compared to WT ONS controls. Error bars represent SD; *n* = 5 independent biological samples; Student's *t* test. (f, g) A‐T ONS cells were transfected with the indicated siRNA for 48hr. (f) Representative Western blot analysis of cGAS and STING of whole cell extracts from siRNA‐transfected cells. α‐tubulin was used as loading control. (g) Total RNA from siRNA‐transfected A‐T ONS patient cells was used to quantify the mRNA expression levels of the indicated SASP genes and normalized to RPLP0 mRNA and compared to siControl. Error bars represent *SD*; *n* = 3 independent experiments; one‐way ANOVA with Tukey's multiple‐comparison post hoc corrections. (h) Representative Western blot analysis of JNK1/2 and ATM of whole cell extracts from siRNA‐transfected WT and A‐T ONS cells. α‐tubulin was used as loading control. (i) WT and A‐T ONS cells were either transfected with the indicated siRNAs, treated with JNK inhibitor (JNKi, SP600125, 20 μM) or MitoQ (100 nM) for ten days. Cells were thereafter assessed for micronuclei formation. Bar graphs show the percentage of micronuclei‐positive cells. Error bars represent *SD*; *n* = 3 independent experiments; one‐way ANOVA with Tukey's multiple‐comparison post hoc corrections

To explore the potential generation of cytoplasmic DNA and senescence hallmarks in A‐T human cells and study its role in neuroinflammation, we employed primary human ONS cells obtained from olfactory mucosa biopsies, comparing ONS cells from five A‐T patients and five healthy controls. Consistent with previous reports in human A‐T skin fibroblasts (Lan et al., 2019), we detected a premature entry into cellular senescence (Figure 1a, b)—as measured by senescence‐associated β‐galactosidase (SA‐β‐gal) activity—and heightened numbers of misshapen nuclei (Figure 1c) in A‐T ONS cells compared to their wild‐type counterparts at identical passage number. This was associated with a concomitant increase in cytoplasmic chromatin content, as measured by elevated numbers of micronuclei (Figure 1d). To further substantiate the direct involvement of ATM in this phenomenon, we exposed primary healthy control dermal fibroblasts to the specific ATM inhibitor KU‐55933 over a three‐day period. Our data showed that acute ATM inhibition also led to a progressive increase in nuclear shape abnormalities (Figure S1a) and micronuclei counts (Figure S1b, c).

Because increased micronuclei have been reported to prime a SASP gene response (Dou et al., 2017), we next quantified mRNA abundance of the SASP genes IL‐6, IL‐8, IL1α and CCL20. Remarkably, all A‐T ONS cells consistently displayed elevated levels of the SASP genes tested as compared to their wild‐type (WT) counterparts (Figure 1e). Since the mechanisms that activate SASP involve a series of events that are connected to DNA damage and the cGAS‐STING pathway (Hartlova et al., 2015), we hypothesized that the elevated levels of micronuclei observed in ONS cells of A‐T patients are recognized by the cGAS‐STING pathway to promote the SASP program. To test this, we stably reduced cGAS or STING expression in WT and A‐T ONS cells via RNA interference (Figure 1f and Figure S1d), and next examined the expression of the SASP genes IL‐6, IL‐8, IL1α and CCL20. Downregulation of either cGAS or STING significantly reduced the expression of SASP genes in A‐T ONS cells (Figure 1g), while SASP gene expression remained unaffected in the WT counterparts (Figure S1e).

Given A‐T’s characteristic phenotype of mitochondrial dysfunction (Valentin‐Vega et al., 2012; Yang et al., 2021) and the fact that dysfunctional mitochondria are known to trigger a reactive oxygen species (ROS)‐JNK retrograde signalling pathway that drives cytoplasmic chromatin formation and the SASP in senescent cells (Vizioli et al., 2020), we tested whether hindering this pathway in A‐T patient ONS cells affects micronuclei accumulation and SASP expression. To directly test this, we exposed WT and A‐T proliferating ONS cells to either concomitant downregulation of JNK1 and JNK2 expression via RNA interference (Figure 1h), the JNK inhibitor SP600125 (Bennett et al., 2001) or the mitochondrial antioxidant MitoQ (Kelso et al., 2001) over a ten‐day period. Interestingly, these three interventions exerted a significant decrease in the number of micronuclei‐positive cells (Figure 1i) and alleviated the expression of the SASP genes tested (Figure S1f) in A‐T ONS cells, while WT counterparts remained affected. These results strongly support the notion that in A‐T patient‐derived cells, micronuclei‐driven SASP expression is markedly dependent on the cytosolic DNA sensing cGAS‐STING pathway and provide functional insights into putative mechanisms of micronuclei generation through a ROS‐JNK signalling in A‐T.

### 2.2 Premature senescence and inflammation in A‐T brain organoids

**FIGURE 2:**
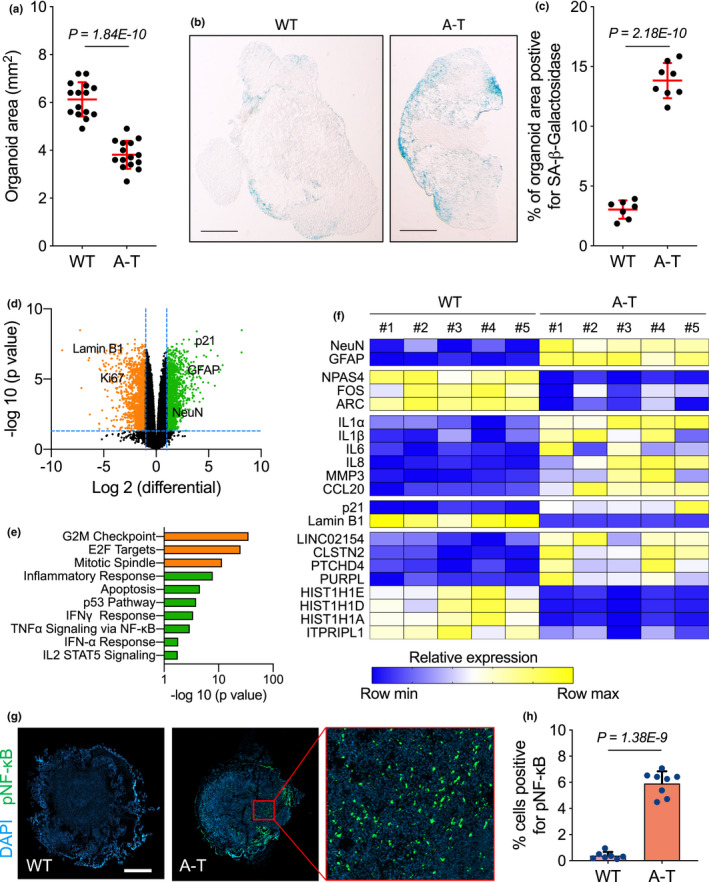
A‐T brain organoids display smaller size and increased levels of senescence and inflammatory marks. (a–h) WT and A‐T brain organoids (BOs) were generated and grown *in vitro* for 3 months and collected for analysis. (a) Each point in the scatter plot shows the organoid size of a single BO. Error bars represent *SD*; *n* = 15 independent BO samples; Student's *t* test. (b) Representative images from quantifications shown in (c). Scale bar, 0.7 mm. (c) SA‐β‐gal assays were performed on wild type (WT) and A‐T BOs. Each point in the scatter plot represents a single BO section analysed. Error bars represent *SD*; *n* = 3 independent experiments; Student's *t* test. (d) Volcano plot showing WT versus A‐T brain organoid differential expression of upregulated (green) and downregulated (orange) genes. (e) Gene Set Enrichment Analysis using ageing hallmark gene sets from the Molecular Signature Database was carried out. The statistically significant signatures were selected (FDR < 0.25) and placed in order of normalized enrichment score, which represents the strength of the relationship between the phenotype and gene signature. Bars indicate the pathways enriched in genes that are downregulated (orange) and upregulated (green) in A‐T BOs as compared to the WT BO group. (f) Total RNA from WT and A‐T BOs were used for RT‐qPCR analysis. B2M mRNA was used as normalizer. *n* = 5 independent biological samples. Exact P values can be found in Figure S2. (g) Immunofluorescence representative images of WT and A‐T BO sections stained for NF‐κB phosphorylated on serine 536 (pNF‐κB). Scale bar, 0.7 mm. (h) Quantification of data presented in (g). Bar graphs show the percentage of pNF‐κB positive cells. Each point in the scatter plot represents a single BO section analysed. Error bars represent *SD*; *n* = 3 independent experiments; Student's *t* test

The brain is one of the major organs to exhibit typical signs of disease in A‐T patients. These include microcephaly, progressive neurodegeneration, chronic inflammation and premature ageing (Rothblum‐Oviatt et al., 2016; Shiloh & Lederman, 2017). Furthermore, A‐T patients likewise exhibit defects in the cortex, such as cortical neuropathology and cerebral white‐matter abnormalities (Ciemins & Horowitz, 2000; Pizzamiglio et al., 2016). To explore whether cGAS‐STING signalling contributes to A‐T neuropathology, we generated cortical brain organoids (BOs) from pluripotent stem cells (PSCs) of WT and A‐T genetic backgrounds. Importantly, droplet digital PCR on genomic DNA of WT and A‐T PSCs confirmed that both cell lines carried wild‐type TP53 sequences (Figure S1g) in regions where mutations are known to affect the ability of p53 to bind to the DNA binding domain and therefore diminish p53‐mediated regulation of apoptosis, cell cycle progression and genomic stability, as previously described (Merkle et al., 2017). Consistent with our data in ONS cells, 3‐month‐old A‐T BOs also displayed elevated levels of micronuclei (Figure S2a‐c). Interestingly, after 3 months of BO development, we observed a significant reduction in organoid size in A‐T patient BOs as compared to their WT counterparts (Figure 2a), a phenomenon that resembles the microcephaly characteristic of a number of genome instability syndromes, including A‐T (Nissenkorn et al., 2011). Given that premature senescence is one of the hallmarks of A‐T, we next performed SA‐β‐gal assays on sections of WT and A‐T organoids. We found that A‐T BOs displayed a significant increase in the proportion of cells that exhibit SA‐β‐gal activity as compared to their control counterparts (Figure 2b, c), suggesting that brain cells in A‐T BOs possess an increased propensity to become senescent. In agreement with the reduction in BO size and increased senescence observed, paired‐end bulk RNA sequencing revealed a set of 3,386 differentially expressed genes in A‐T BOs (Figure 2d and Figure S2d) that were enriched for many of the known ageing pathways identified in the hallmark gene set collection within the Molecular Signatures Database, including several pro‐inflammatory signatures (Figure 2e). Real‐time PCR experiments further confirmed increased expression of reported cGAS‐STING‐dependent SASP genes (MMP3, IL1β, IL‐6, IL‐8, IL1α and CCL20) (Figure 2f and Figure S2f).

**FIGURE 3:**
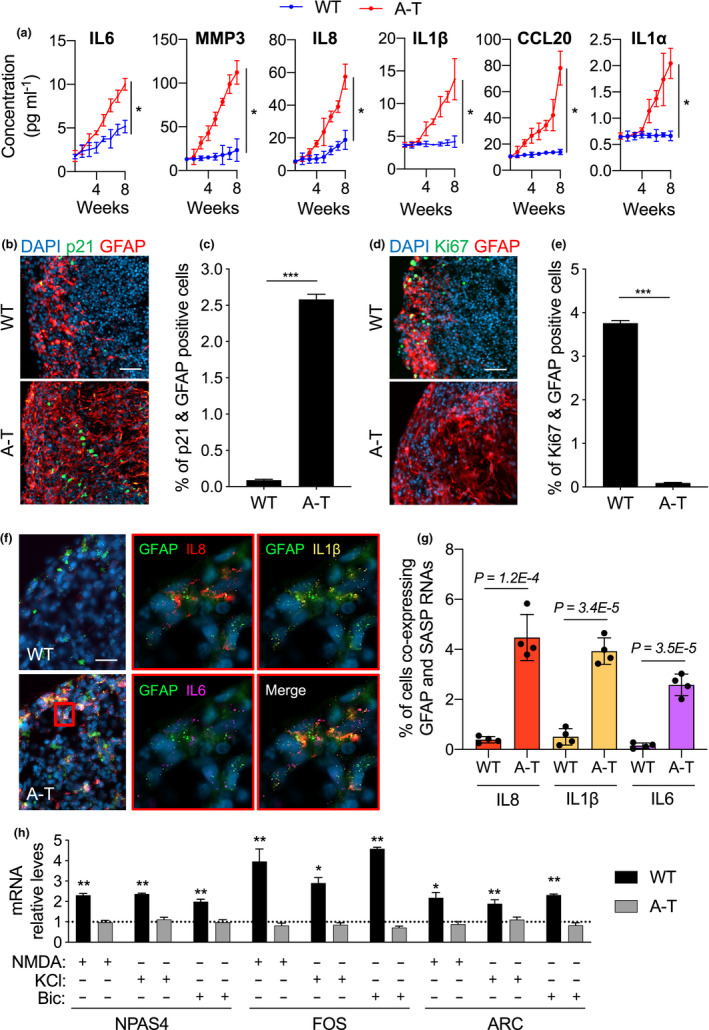
A‐T brain organoids secrete enriched levels of pro‐inflammatory factors and fail to transcribe IEGs upon neuronal excitatory input. (a) Culture media from WT and A‐T BOs was collected once a week for 2 months and used to quantify the secreted levels of the indicated SASP proteins. *n* = 3 independent experiments. *p < 0.05; two‐way ANOVA with concentration and time used as variables. (b‐h) WT and A‐T Brain organoids (BOs) were generated and grown *in vitro* for 3 months and collected for analysis. (b) Representative images of BO sections stained for p21 (green) and GFAP (red). Scale bar, 100 µm. (c) Quantification of data presented in b. Bar graphs show the percentage of p21 and GFAP positive cells ±95% confidence interval. *n* = 3 independent biological samples; at least 100,000 cells per sample have been analysed; ***p < 0.001, chi‐squared test. (d) Representative images of BO sections stained for Ki67 (green) and GFAP (red). Scale bar, 100 µm. (e) Quantification of data presented in d. Bar graphs show the percentage of Ki67 and GFAP positive cells ±95% confidence interval. *n* = 3 independent biological samples; at least 100,000 cells per sample have been analysed; ***p < 0.001, chi‐squared test. (f) Representative images of RNAscope *in situ* hybridization on BO sections for GFAP (green), IL‐8 (red), IL1B (yellow) and IL‐6 (magenta). Cell nuclei were stained with DAPI (blue). Scale bars, 30 μm. (g) Quantification of data presented in f. Bar graphs show the percentage of cells that are simultaneously labelled by GFAP and the indicated SASP RNAscope HiPlex probes. Error bars represent *SD*; *n* = 4 independent biological samples; Student's *t* test. (h) 3‐month‐old WT and A‐T BOs were treated with NMDA (100 µM), KCl (55mM) and Bicuculline (50 µM) for 30 min. Immediately after, BOs were collected for RNA extraction. RT‐qPCR analysis of the indicated IEGs was performed, and RPLP0 mRNA was used as normalizer. Bar graphs show fold change of IEG levels in stimulated organoids relative to untreated (depicted by a grid line). Error bars represent *SD*; *n* = 3 independent experiments. *p<0.05, **p<0.01, Student's *t* test

Given that transcription of the SASP upon GAS and STING activation is mediated by phosphorylation of the transcription factors NF‐κB on serine 536 (pNF‐κB) and IRF3 on serine 386 (pIRF3), we next examined the *in situ* accumulation of pNF‐κB and pIRF3 in WT and A‐T BO sections. Notably, A‐T BOs displayed significantly higher levels of pNF‐κB (Figure 2g, h) and pIRF3 (Figure S2g, h) as compared to the WT counterparts. To examine whether the detected SASP transcriptional changes were also observed at the protein level, we next monitored the secretion of SASP protein factors in BO media, which we harvested once a week for 2 months. Consistent with the SASP RNA expression data and SA‐β‐gal experiments, A‐T BOs displayed a significantly higher secretion of SASP proteins compared to their WT counterparts (Figure 3a), a result indicative of a putative role of SASP factors in inducing paracrine senescence, as previously described (Acosta et al., 2013; Nelson et al., 2012). Consistent with the increased abundance of senescent cells that exhibit SA‐β‐gal activity, our RNA‐seq and real‐time PCR data revealed increased mRNA levels of the canonical senescence marker p21 (CDKN1A) and reduced expression of Lamin B1 (LMNB1, Figure 2d and f and Figure S2e) in A‐T BOs as compared to their WT counterparts. In addition, A‐T BOs consistently expressed higher levels of other genes typically induced (Casella et al., 2019) in senescent cells (LINC02154, CLSTN2, PTCGD4 and PURPL) and expressed lower levels of genes generally reduced (Casella et al., 2019) in senescent cells (HIST1H1E, HIST1H1D, HIST1H1A and ITPRIPL1); further substantiating the increased cellular senescence phenotype associated with BOs derived from A‐T iPSCs (Figure 2f and Figure S2i). Interestingly, A‐T BOs displayed premature neuronal and astrocytic differentiation as compared to WT controls, as indicated by the early increase in RNA expression and protein expression of the mature neuronal marker NeuN and the astrocyte marker GFAP in 3‐month‐old A‐T BOs (Figure 2d and f and Figure S3a–d), a phenomenon that appears to be conserved among genome instability syndromes, such as Nijmegen Breakage Syndrome (Martins et al. 2020).

Previous studies reported that senescent astrocytes play a role in driving the loss of functional neurons (Gosselin & Rivest, 2018), thus we next examined the extent of astrocyte senescence in WT and A‐T BOs. We found a >28‐fold increase in cells co‐expressing p21 and the astrocyte marker GFAP in the A‐T BOs as compared to WT controls (Figure 3b and c). Interestingly, the presence of senescent oligodendrocyte progenitor cells (OPC)—which previously were found to drive amyloid‐beta pathology in a model of Alzheimer's disease (Zhang et al., 2019)—was hardly manifest in A‐T BOs (Figure S3e). In fact, senescent OPCs, as measured by cells co‐staining for p21 and the OPC marker NG2, accounted for <2% of the total number of senescent cells in the A‐T BOs (Figure S3f). Conversely, >52% of p21‐positive cells exclusively belonged to astrocyte populations (Figure S3f). Concomitantly, A‐T BOs exhibited a strong reduction in astrocytes positive for the proliferation marker Ki67 as compared to WT (Figure 3d, e), further indicating a strong astrocyte‐specific senescence phenotype in A‐T BOs. To test whether the senescent astrocyte phenotype in A‐T BOs led to aberrant SASP expression, we assessed by RNA *in situ* hybridization the co‐localization of SASP mRNAs with the astrocyte transcript GFAP (Figure 3f). Importantly, we observed a significant increase in the number of astrocytes expressing the SASP genes IL‐8, IL1β and IL‐6 (Figure 3g). Given that cellular senescence and consequent SASP detrimental secretome is reported to induce degeneration in neurons (Bussian et al., 2018; Chinta et al., 2018), we next examined whether the accumulation of senescent astrocytes impacted the functionality of neurons in A‐T BOs, through quantification of mRNA expression of the Immediate Early Genes (IEGs) FOS, NPAS4 and ARC, as previously reported (Madabhushi et al., 2015). Interestingly, A‐T BOs displayed significantly lower expression of IEGs than WT BOs (Figure 2f and Figure S3g) that could not be upregulated by exposure to excitatory stimulation with KCl, NMDA or Bicuculline, whereas healthy control BOs showed a robust expression of IEGs that were further increased by excitatory stimulation (Figure 3h). Altogether, these results demonstrate that A‐T BOs exhibit a significant accumulation of senescent astrocytes that promote a pro‐inflammatory environment, which in turn results in neurons that display dysfunctional neuronal activity.

### 2.3 cGAS or STING inhibition reduces A‐T brain organoid senescence

Since the above data support the putative role for the cGAS‐STING pathway in driving premature senescence in A‐T BOs, we next examined the long‐term consequence of premature senescence on neuronal survival. We then generated BOs and allowed them to age for up to 6 months. Quantification of cells labelled with NeuN in 6‐month‐old BOs revealed a significant decrease in the number of mature neurons in A‐T BOs compared to the WT counterparts (Figure S3h), indicative of either impaired neuronal development or accelerated neurodegeneration in A‐T organoids.

**FIGURE 4:**
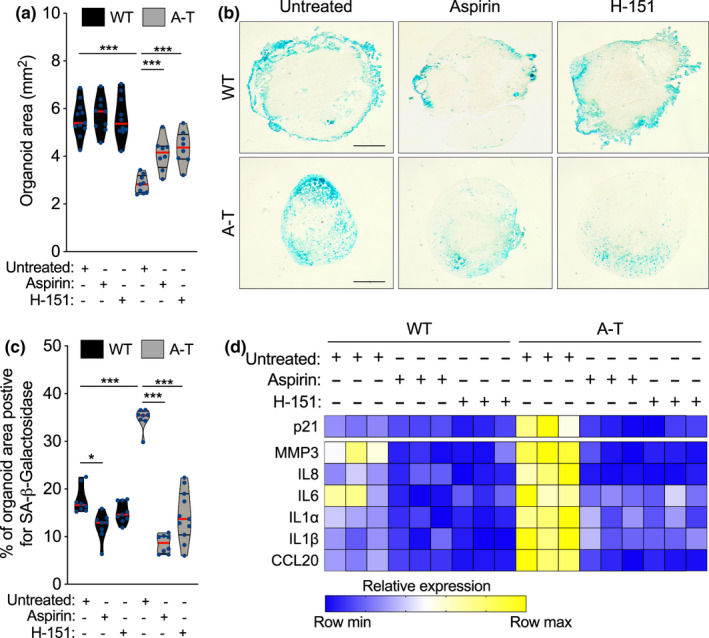
Inhibition of cGAS and STING reduces tissue degeneration and cellular senescence in brain organoids. (a–e) WT and A‐T BOs where treated for one month with aspirin (4mM) or H‐151 (3 µM) starting at 5 months of development. (a) Each point in the scatter plot shows the organoid size of a single BO. At least 8 organoids per condition were analysed. ***p < 0.001; one‐way ANOVA with Tukey's multiple‐comparison post hoc corrections. (b) Representative images from quantifications shown in c. Scale bar, 0.7 mm. (c) SA‐β‐gal assays were performed on WT and A‐T BOs with the indicated treatments. Each point in the scatter plot represents a single BO section analysed. *n* = 3 independent experiments. *p < 0.05; ***p < 0.001; one‐way ANOVA with Tukey's multiple‐comparison post hoc corrections. (e) Total RNA from WT and A‐T BOs were used for RT‐qPCR analysis. B2M mRNA was used as normalizer. *n* = 3 independent biological samples. Exact P values can be found in Figure S4

To study the contribution of the cGAS and STING pathways to this detrimental neuronal phenotype in A‐T BOs, we next investigated whether we could ameliorate SASP and senescence with previously reported specific cGAS and STING inhibitors (Dai et al., 2019; Haag et al., 2018). To this end, we treated BOs weekly with the cGAS inhibitor aspirin (Dai et al., 2019) or the STING inhibitor H‐151 (Haag et al., 2018) for a period of one month prior to reaching the sixth month of BOs development (Figure S4a). Excitingly, we observed that incubation with either of these inhibitors significantly rescued the loss of organoid size in A‐T BOs, while WT’s size remained unaltered (Figure 4a). Furthermore, both cGAS and STING inhibition significantly reduced the abundance of cells with SA‐β‐gal activity in A‐T BOs to levels comparable to their control counterparts, (Figure 4b, c), and simultaneously led to a concomitant reduction in p21 and SASP expression in A‐T BOs (Figure 4d and Figure S4b–h). Taken together, these results strongly indicate that inhibition of cGAS‐STING signalling in AT‐BOs decreases senescence phenotypes, reduces inflammation and has beneficial effects on brain tissue homeostasis.

### 2.4 Inhibition of cGAS‐STING improves A‐T neuropathology

**FIGURE 5:**
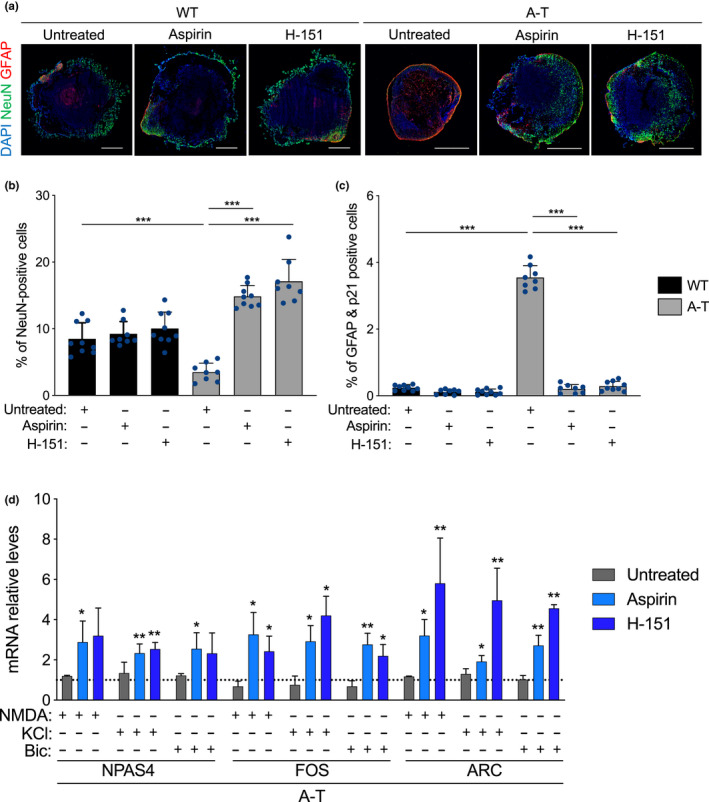
cGAS and STING inhibition prevents neuronal loss and rescues the transcriptional activation of IEGs upon neuronal excitatory input. (a‐d) WT and A‐T BOs were treated for one month with aspirin (4mM) or H‐151 (3 µM) starting at 5 months of development. (a) Immunofluorescence representative images of BO sections stained for NeuN (green) and GFAP (red). Scale bar, 0.7 mm. (b,c) BO sections stained for NeuN (b) and GFAP & p21 (c) were quantified. Each point in the scatter plot represents a single BO section analysed. Error bars represent *SD*; *n* = 4 independent experiments. ***p < 0.001; one‐way ANOVA with Tukey's multiple‐comparison post hoc corrections. (d) A‐T BOs were treated with NMDA (100 µM), KCl (55 mM) and Bicuculline (50 µM) for 30 min. Immediately after, BOs were collected for RNA extraction. RT‐qPCR analysis of the indicated IEGs was performed, and B2M mRNA was used as normalizer. Bar graphs show fold induction of IEG levels in stimulated organoids relative to untreated (no neuronal‐stimulating drug, depicted by a grid line). Error bars represent *SD*; *n* = 3 independent experiments. *p < 0.05; **p < 0.01, Each bar was compared to grid line values. Student's *t* test

Encouraged by the reduction in senescence phenotypes in A‐T BOs brought about by aspirin or H‐151 treatment, we next investigated whether inhibition of the cGAS‐STING pathway would also improve the compromised neuronal survival and function observed in A‐T BOs. To this end, we first treated WT and A‐T BOs with either inhibitor for one month, similarly to the treatment schedule described above, and subsequently stained for phosphorylated STING on serine 366 (pSTING) to prove the efficacy of both inhibitors in disrupting the cGAS‐STING signalling pathway. Notably, A‐T BOs displayed significantly higher STING activation compared to WT counterparts, as measured by the percentage of cells positive for pSTING (Figure S5a,b). In addition, both aspirin and H‐151 exerted a decline in the number of BO cells positive for pSTING (Figure S5a,b), though the effects were more evident upon H‐151 administration, and in the A‐T BOs given their stronger STING activation as compared to WT. Strikingly, treatment of A‐T BOs with either inhibitor significantly improved the survival of neurons compared to A‐T untreated BOs, as measured by the number of NeuN positive cells (Figure 5a, b). Interestingly, cGAS and STING inhibition exerted a marginally better neuronal survival in A‐T as compared to WT, which may be explained by the alleviation of a restrictive environment instigated by a significantly heightened senescence phenotype in A‐T as compared to WT BOs. Specifically, A‐T BOs showed increased senescent astrocytes compared to their control counterparts as measured by the percentage of cells that co‐stained for p21 and GFAP markers (Figure 5c). Most importantly, the number of senescent astrocytes was drastically reduced by prolonged inhibition of the cGAS‐STING axis (Figure 5c and Figure S5c), further indicating a detrimental role for senescent astrocytes in driving neurodegeneration in A‐T.

We next examined whether this strong decrease in senescent astrocytes impacts the functionality of neurons in A‐T BOs, through quantification of mRNA expression of IEGs. Consistent with the rescue in neuronal counts (Figure 5b), cGAS or STING inhibitors significantly restored the capacity of neurons within A‐T BOs to respond to excitatory inputs while the untreated counterparts again failed to transcribe IEGs upon stimulation (Figure 5d). Notably, both untreated and inhibitor‐treated WT BOs experienced a comparable transcriptional activation of IEGs upon neuronal stimulation (Figure S5d). In addition, aspirin or H‐151 treatments did not significantly alter the number of NeuN positive cells in WT BOs (Figure 5a, b), suggesting that neither cGAS nor STING inhibition has detrimental effects on healthy WT neurons in this model system. In summary, these results demonstrate that inhibition of cGAS or STING with aspirin or H‐151 respectively, mitigates astrocyte senescence in A‐T BOs, resulting in improved neuronal function and survival and a significant reduction in brain organoid inflammatory signatures.

## 3 DISCUSSION

Ageing in the brain is characterized by increased inflammation, a decline in DNA repair, accumulation of senescent cells, and these each represent major risk factors for neurodegenerative diseases (Hou et al., 2019). A‐T is a genome instability syndrome (Taylor et al., 2019) that displays several hallmarks of premature ageing (Shiloh & Lederman, 2017), including brain degenerative changes and neuronal loss (Rothblum‐Oviatt et al., 2016). Importantly, inflammation is a major component of this disease, and both fibroblasts from A‐T patients and Atm null murine models exhibit accumulation of cytosolic DNA, which in turn is responsible for activating the canonical cGAS‐STING axis (Hartlova et al., 2015; Lan et al., 2019). However, the contribution of this A‐T self‐DNA‐induced SASP induction to brain ageing and degeneration remains uncharacterized. This is for a large part due to the fact that until recently, no experimental approach allowed modelling of A‐T neurological deficiencies directly from patient cells. BOs have become an invaluable model platform for human brain disease modelling as they accurately model complex neuronal networks and reproducibly recapitulate the cell diversity of the developing human cerebral cortex (Velasco et al., 2019). This is particularly relevant for the study of A‐T neuropathology, as mouse models of A‐T generally fail to exhibit a number of prominent neurological abnormalities observed in A‐T patients, one possible reason may be partly attributed to the early onset of lymphoid tumours and consequent premature death of the mice (Lavin, 2013). Here, we used human A‐T patient iPSC‐derived BOs to determine the contribution of senescent cells and consequent activation of the cGAS‐STING pathway in A‐T neuropathology.

In this study, we first showed that primary ONS cells from nasal biopsies of five A‐T patients consistently display nuclear shape abnormalities and increased micronuclei with concomitant elevated pro‐inflammatory SASP levels. Importantly, delivery of siRNAs against cGAS and STING in A‐T ONS cells significantly reduced the expression of a number of SASP genes, indicating that the cGAS‐STING pathway is required for driving inflammation in human A‐T ONS cells.

Furthermore, we showed that human BOs of A‐T accumulate increased numbers of senescent cells that are largely comprised of astrocyte populations. While senescent OPCs and microglia are reported to contribute to age‐related neurodegeneration in the mouse brain (Ogrodnik et al., 2021; Zhang et al., 2019), our data suggest that OPCs undergo minimal cellular senescence onset in A‐T BOs. As regards to microglia, we did not characterise their role in this model as the BO protocol we utilized relies on dual‐SMAD inhibition to rapidly induce neuroectoderm formation, which limits the generation of mesoderm‐derived progenitor cells and subsequent differentiation into mature microglia. Importantly, the enriched population of senescent astrocytes observed in A‐T BOs induces a pro‐inflammatory response in a cGAS‐STING‐dependent manner, which in turn impairs correct brain organoid development, results in decreased BOs’ size, and compromises immediate early gene transcriptional activation upon neuronal stimulation. Remarkably, A‐T BOs recapitulate the microcephaly phenotype previously reported in A‐T patients (Nissenkorn et al., 2011), and chemical inhibition of cGAS and STING via aspirin (Dai et al., 2019) or H‐151 (Haag et al., 2018), respectively, rescued both BO size defects and entry into cellular senescence. Furthermore, cGAS‐STING inhibition prevented neuronal loss and rescued neuronal function in A‐T BOs, suggesting a detrimental impact of senescent astrocytes on neuronal homeostasis in this disease. This is consistent with recent reports supporting the notion that senescent astrocytes promote neuronal toxicity and degeneration (Chinta et al., 2018). These results demonstrate the central and necessary contribution of cGAS‐STING‐driven inflammation in the senescent phenotypes of A‐T neuropathology. Conceptually, our results in BOs indicate that not only senescence *per se* but also the resulting inflammatory SASP activation of senescent cells is responsible for the detrimental neuropathogenic effects observed in A‐T. Moreover, our data demonstrate for the first time that the so‐called senomorphics—pharmacological agents designed to suppress the SASP (Di Micco et al., 2020)—are effective in reducing the phenotypes associated with cellular senescence in human brain models. Indeed, this family of senomorphic approaches designed to reduce or alter the SASP have shown promise *in vivo* (Aguado et al., 2019; Dai et al., 2019; Haag et al., 2018; Milanovic et al., 2018), suggesting that novel therapeutic compounds targeting the drivers of SASP, including cGAS and STING, could greatly advance this area of research.

It is worth highlighting that these conclusions may have impact beyond A‐T neuropathology. A number of neurodegenerative disorders, including Alzheimer's, Parkinson's disease, Werner's and Down's syndrome, constitute a collection of conditions that are associated with aberrantly elevated micronuclei frequencies (Migliore et al., 2011), but the potential neuropathogenic contribution of cGAS‐STING activation consequent to increased cytosolic DNA remains to be investigated in these conditions. While we recognize that the aspirin concentration used in this study (4 mM) exceeds the tolerable dose in humans (as reviewed in Elkon, 2019), a number of additional more specific cGAS and STING inhibitors have been reported, including the small molecule inhibitor H‐151 used in this study, that may offer clinically translatable opportunities to selectively inhibit this senescence‐driven pro‐inflammatory pathway characteristic of A‐T. Here, we have provided evidence that the use of aspirin and H‐151 in preventing cGAS and STING activity are efficient ways to inhibit senescent astrocyte‐driven inflammation in a relevant brain organoid model, and we have validated cGAS‐STING inhibition as a potent therapeutic target for A‐T neuropathology and potentially for other neurological diseases associated with premature ageing and significant self‐DNA‐induced SASP activation.

## 4 METHODS

### 4.1 Olfactory biopsies

As previously described (Stewart et al., 2013), nasal biopsies were collected from controls and A‐T patients. Biopsies were obtained with informed consent from the parents in accordance with the Australian NHMRC Code of Practice for Human Experimentation and approved by the Queensland Children's Health Services (RCH) and Griffith University Human Ethics Committees. A‐T patients were diagnosed after attendance at the A‐T Clinic, University of Queensland Centre for Clinical Research, Brisbane.

### 4.2 Cell culture

Human mortal fibroblasts (ATCC, CRL‐2429) were grown in DMEM, supplemented with 20% foetal bovine serum (FBS), 1:100 Glutamax (Invitrogen, cat. #35050‐038), 1:100 minimum essential media‐nonessential amino acids (MEM‐NEAA, Sigma, cat. #M7145) and 1 mM sodium pyruvate. Human H9 (WA09) cells were obtained from WiCell with verified normal karyotype and contamination‐free. iPSCs were generated from ONS cells (GRIDD Neuro Bank ID # 906110003) of one A‐T patient using the non‐integrating, self‐replicating RNA reprogramming vector ReproRNA‐OKSGM (Stemcell Technologies, cat. # 05930) according to manufacturer's instructions. The generated iPSC line (A‐T HL; unique identifier AIBNi014‐A) was verified pluripotent and contamination‐free. All human pluripotent stem cell lines were routinely tested and confirmed negative for mycoplasma (MycoAlert, Lonza). For JNK and mitochondrial ROS inhibition, cells were treated with 20 μM SP600125 (Abcam, cat. # ab120065) and 100 nM MitoQ (MCE, cat. # HY‐100116A), respectively, for 10 days. For ATM inhibition, cells were treated with 10 µM KU‐55933 (Enzo, cat. # CHM150) for 3 days. Wild‐type and A‐T patient‐derived human ONS cells were grown in DMEM, supplemented with 20% FBS, 1:100 Glutamax, 1:100 MEM‐NEAA and 1 mM sodium pyruvate. Informed consent has been obtained for these cells, which were donated to Griffith Institute for Drug Discovery (GRIDD) by patient families to be used for research on ataxia‐telangiectasia. Samples, part of NeuroBank, a larger collection of ONS cells from patients with neurological conditions, belong to GRIDD at Griffith University located in the N75 building and stored in liquid nitrogen vapour. All cells were grown at 37℃, 5% CO_2_.

### 4.3 Brain organoid generation and culture conditions

Pluripotent stem cells were maintained in mTeSR media (STEMCELL Technologies, cat. #85850). WT BOs were generated from human H9 (WA09) cells and A‐T BOs from A‐T patient‐derived iPSCs. On day 0 of organoid culture, H9 and A‐T iPS cells were dissociated with Accutase (Life Technologies, cat. #00‐4555‐56) and seeded at a density of 15,000 cells per well on 96‐well low‐attachment U‐bottom plates (Sigma, cat. #CLS7007) in mTeSR and 10 μM Y‐27632, a Rho‐associated protein kinase inhibitor (VWR, cat. #688000‐5). The 96‐well plates were then spun at 330 g for 5 min to aggregate the cells and make spheroids. The spheroids were fed every day for 5 days in media containing Dulbecco's modified eagle medium (DMEM)/F12 (Invitrogen, cat. #11330‐032), Knock‐out serum (Invitrogen, cat. #11320‐033), 1:100 Glutamax, 1:200 MEM‐NEAA supplemented with dual‐SMAD inhibitors: 2 μM Dorsomorphin (StemMACS, cat. #130‐104‐466) and 2 μM A‐83‐01 (Lonza, cat. #9094360). On day 6, half of the medium was changed to induction medium containing DMEM/F12, 1:200 MEM‐NEAA, 1:100 Glutamax, 1:100 N2 supplement (Invitrogen, cat. #17502048), 1 μg/ml heparin (Sigma, cat. # H3149) supplemented with 1 μM CHIR 99021 (Lonza, cat. #2520691) and 1 μM SB‐431542 (Sigma, cat. # S4317). From day 7, complete media change was done with induction media followed by everyday media changes in induction media for the next 4 days. On day 11 of the protocol, spheroids were transferred to 11 μl‐droplets of Matrigel (Corning, cat. # 354234) on a sheet of Parafilm with small 3 mm dimples. These droplets were allowed to gel at 37℃ for 40 min and were subsequently removed from the Parafilm and transferred to and maintained in low‐attachment 24‐well plates (Sigma, cat. #CLS3473) containing induction medium for the following 5 days. From day 16, the medium was then changed to organoid medium containing a 1:1 mixture of Neurobasal medium (Invitrogen, cat. #21103049) and DMEM/F12 medium supplemented with 1:200 MEM‐NEAA, 1:100 Glutamax, 1:100 N2 supplement, 1:50 B27 supplement (Invitrogen, cat. #12587010), 1% penicillin‐streptomycin (Sigma, cat. #P0781), 50 μM 2‐mercaptoethanol and 0.25% insulin solution (Sigma, cat. #I9278). Media was changed three times per week with organoid medium. Organoids were maintained in organoid media until the end of experiments, as indicated.

### 4.4 RNA isolation

Total RNA from cultured cells and brain organoids was extracted with RNeasy Mini Kit (Qiagen) for mRNA detection, according to the manufacturer's instructions. Organoid tissue was homogenized with a TissueLyser II (Qiagen) at 20 Hz for 25 seconds. RNA integrity of cultured cell and brain organoids was evaluated by analysis on the 2100 Bioanalyzer RNA 6000 Pico Chip kit (Agilent) using the RNA Integrity Number (RIN). RNA samples with a RIN >7 were considered of high enough quality for real‐time quantitative PCR, and transcriptomic library construction and RNA sequencing according to the manufacturer's instructions.

### 4.5 RNA Sequencing and bioinformatics analyses

Before mRNA sequencing, ribosomal RNA was depleted with the Ribo‐Zero rRNA Removal Kit (Illumina). Transcripts were sequenced at Novogene Ltd (Hong Kong) using TruSeq stranded total RNA library preparation and Illumina NovaSeq 150bp paired‐end lane. Sequencing provided >100 million reads per sample. FastQC (v.11.5) was used to check quality on the raw sequences before analysis to confirm data integrity. Trimming was performed using Trimmomatic (v.0.38), and reads were mapped against the human reference genome (release hg38) using bwa (v.0.74). Differential gene expression (DGE) analysis was performed using Bioconductor R packages. Limma‐voom was used to estimate the mean‐variance relationship of the log‐counts, obtain a precision weight for each observation and perform DGE analysis with empirical Bayes pipeline. To ensure high quality of the count table, the raw count table generated by featureCount was filtered for subsequent analysis. First, the raw count was transformed to count‐per‐million (cpm) value. Then, genes with 0 cpm value across all ten samples were excluded from DGE analysis. The raw count contained 38557 genes and 25391 passed the filtering process and were used for statistical analysis. A total of 13166 genes failed to meet the filtering criteria. Benjamini–Hochberg multiple testing correction was applied to the DGE to obtain the adjusted P value for all genes. Genes were defined as significantly up‐ or downregulated when A‐T brain organoids presented a *p* value <0.05 compared to wild‐type brain organoids. Gene expression was analysed by comparison between 5 WT and 5 A‐T brain organoids.

### 4.6 Real‐time quantitative PCR

1 μg of total RNA was reverse transcribed using SuperScript VILO cDNA Synthesis Kit. A volume corresponding to 5 ng of initial RNA was employed for each real‐time PCR reaction using SsoFast EvaGreen Supermix (Bio‐Rad) or PowerUp SYBR Green Master Mix (Applied Biosystems) on a Bio‐Rad CFX96 Touch Real‐Time PCR detection system. Each reaction was performed in triplicate. Human beta‐2‐microglobulin (B2M) or ribosomal protein P0 (RPLP0) was used as control transcripts for normalization. Primers sequences (5′‐3′ orientation) are listed in Table S1.

### 4.7 Droplet Digital PCR

Genomic DNA from H9 and A‐T pluripotent stem cells was extracted with DNeasy Blood & Tissue Kit (Qiagen) to test the allelic fraction of the four most distinct TP53 mutations by droplet digital PCR (ddPCR) as previously described (Merkle et al., 2017). Briefly, each ddPCR reaction utilized a custom TaqMan assay (IDT)—which consisted of a primer pair and two 5′ fluorescently labelled probes with a 3′ quencher for either reference or mutant base—for the detection of the four TP53 variants (Table S2). Genomic DNA from both cell lines was analysed by ddPCR according to the manufacturer's protocol (Bio‐Rad) on a QX200 Droplet Digital PCR System.

### 4.8 siRNA Transfection

ON‐TARGETplus SMARTpool short interfering RNA (siRNA) oligonucleotides (Dharmacon) were used at a final concentration of 20 nM. siRNA transfections were carried out with Lipofectamine RNAiMAX (Invitrogen) according to the manufacturer's instructions.

Sequences were as follows (5′‐3′ orientation):

siControl (non‐targeting pool): UGGUUUACAUGUCGACUAA; UGGUUUACAUGUUGUGUGA; UGGUUUACAUGUUUUCUGA; UGGUUUACAUGUUUUCCUA
sicGAS: GAAGAAACAUGGCGGCUAU; AGGAAGCAACUACGACUAA; AGAACUAGAGUCACCCUAA; CCAAGAAGGCCUGCGCAUU
siSTING: UCAUAAACUUUGGAUGCUA; CGAACUCUCUCAAUGGUAU; AGCUGGGACUGCUGUUAAA; GCAGAUGACAGCAGCUUCU
siJNK1: GCCCAGUAAUAUAGUAGUA; GGCAUGGGCUACAAGGAAA; GAAUAGUAUGCGCAGCUUA; GAUGACGCCUUAUGUAGUG
siJNK2: GAUUGUUUGUGCUGCAUUU; GGCUGUCGAUGAUAGGUUA; AGCCAACUGUGAGGAAUUA; UCGUGAACUUGUCCUCUUA

### 4.9 Immunoblot

Cells were lysed in Laemmli buffer (2% SDS, 10% glycerol, 60 mM Tris HCl pH 6.8). 40 μg of whole cell lysates was resolved by SDS polyacrylamide gel electrophoresis. Proteins were transferred to a nitrocellulose membrane and subsequently blocked in 5% milk in TBST (Tris‐buffered saline containing 0.1% Tween‐20). Primary antibodies were incubated overnight at 4℃ and horseradish peroxidase‐conjugated secondary antibodies were incubated for 1 hour at RT. Image acquisition was performed with a ChemiDoc Imager (Bio‐Rad).

### 4.10 Organoid sectioning and immunofluorescence

Organoids were fixed in 4% paraformaldehyde (PFA) for 1 h at RT and washed with phosphate‐buffered saline (PBS) three times for 10 min each at RT before allowing to sink in 30% sucrose at 4℃ overnight and then embedded in OCT (Agar Scientific, cat. #AGR1180) and cryosectioned at 14 μm with a Thermo Scientific NX70 Cryostat. Tissue sections were used for immunofluorescence and for the senescence‐associated β‐galactosidase assay. For immunofluorescence, sections were blocked and permeabilized in 0.1% Triton X‐100 and 3% bovine serum albumin (BSA) in PBS. Sections were incubated with primary antibodies overnight at 4℃, washed and incubated with secondary antibodies for 60 min at RT. 0.5 μg ml^−1^ DAPI (Sigma, cat. #D9564) was added to secondary antibody to mark nuclei. Secondary antibodies labelled with Alexafluor 488, 568 or 647 (Invitrogen) were used for detection.

### 4.11 Immunofluorescence for cultured cells

Cells were fixed with 4% PFA solution. After incubation with blocking solution (0.5% BSA and 0.2% Gelatin from cold water fish skin in PBS) for 1 h at RT, cells were stained with primary antibody for 1 h at RT or overnight at 4℃, washed and incubated with secondary antibodies for 45 min at RT. Nuclei were stained with DAPI (0.5 μg/ml). Samples were mounted in mowiol solution (Calbiochem).

### 4.12 RNA in situ hybridization

RNA *in situ* hybridization (ISH) was performed using an RNAscope HiPlex Kit (Advanced Cell Diagnostics) with detection probes against GFAP, IL1B, IL‐8 and IL‐6 mRNAs following directions of the manufacturer. Brain organoids for RNA ISH were sectioned as described above and sections were consecutively exposed to target retrieval conditions followed by digestion with protease, hybridization with target probes, amplification and labelling with fluorophores. Samples were counterstained with DAPI to visualize nuclei, and slides were imaged with a Zeiss AxioScan Z1 Fluorescent Imager.

### 4.13 Senescence‐associated β‐galactosidase assay (SA‐β‐Gal)

Cells were washed in PBS, fixed for 10 min in 4% PFA, washed and incubated at 37℃ (in the absence of carbon dioxide) with fresh SA‐β‐Gal stain solution (pH 6.0): Potassium ferricyanide 5 mM, potassium ferrocyanide 5 mM, sodium dihydrogen phosphate 0.4 M, sodium hydrogen phosphate 92 mM, sodium chloride 150 mM, magnesium dichloride 2 mM and 1 mg/ml of 5‐bromo‐4‐chloro‐3‐indolyl‐β‐D‐galactopyranoside. Staining was evident in 2–4 h and maximal in 12–16 h.

### 4.14 Imaging and analysis

Immunofluorescence images were acquired using a multiphoton confocal Leica TCS SP8MP fluorescence microscope or a Zeiss AxioScan Z1 Fluorescent Imager. For organoid stainings, the number of positive cells per organoid for senescence, astrocyte and neuronal markers tested was analysed by the imaging software CellProfiler, using the same pipeline for each sample in the same experiment. To analyse image circularity, processing of each confocal microscope high‐resolution image consisted of a consecutive series of algorithms implemented as plugins in the freely available software ImageJ (http://imagej.nih.gov/ij/. The outlines of segmented nuclei are determined using edge detection algorithms based on differential brightness cut‐offs. Circularity indexes range from 1.0 (representing a perfect circle) to 0 (representing a straight line).

### 4.15 Measurement of organoid‐secreted proteins

Media concentrations of IL‐1α, IL‐1β, IL‐6, IL‐8, MMP3 and CCL20 were assessed using a custom‐made magnetic luminex screening assay (R&D Systems) according to the manufacturer's instructions and read on a MAGPIX instrument (Luminex Corp).

### 4.16 Antibodies

Anti‐cGAS (Cell Signalling, 15102, 1:1000); anti‐STING (Cell Signalling, 13647, 1:1000); anti‐pSTING (Cell Signalling, 40818, 1:200); anti‐Tubulin (Sigma‐Aldrich, T5168, 1:2000); anti‐p21 (Cell Signalling, 2946S, 1:400); anti‐Ki67 (ThermoFisher Scientific, PA1‐21520, 1:100); anti‐NeuN (Millipore, ABN78, 1:1000); anti‐NeuN (Millipore, MAB377, 1:1000); anti‐GFAP (Sigma‐Aldrich, G3893, 1:1000); anti‐GFAP (Agilent, Z0334, 1:2000); anti‐pNF‐κB (Cell Signalling, 3033, 1:500); anti‐pIRF3 (Cell Signalling, 37829, 1:400); anti‐JNK (R&D Systems, AF1387SP, 1:1000); anti‐NG2 (Sigma‐Aldrich, AB5320, 1:200); anti‐Mouse IgG (Jackson ImmunoResearch, 715‐165‐151, 1:500); anti‐rat IgG (Jackson ImmunoResearch, 712‐605‐153, 1:500); anti‐rabbit IgG (Jackson ImmunoResearch, 711‐165‐152, 1:500); HRP anti‐mouse IgG (Cell Signalling, 7076S, 1:10000); HRP anti‐rabbit IgG (Cell Signalling, 7074S, 1:10,000).

### 4.17 Statistical analysis

Results are shown as mean ± standard error of the mean (SEM) or standard deviation (SD) or as percentages ±95% confidence interval as indicated. *p* Value was calculated by the indicated statistical tests, using Prism software. In figure legends, n indicates the number of independent experiments.

## CONFLICT OF INTEREST

The authors declare no competing interests.

## AUTHOR CONTRIBUTIONS

H.C. generated human brain organoids and data on Figures 2a, 3b,c and S3a‐d. C.G.I generated the results in Figure 1a,b. M.S. performed stainings shown in Figure 3d,e. H.L. performed the reprogramming of A‐T patient ONS cells to iPSCs. A.M.S. provided the ONS cells. J.A. generated data in all remaining figures;.J.A. and E.W. designed the study and wrote the paper. All authors edited the paper.

## Supporting information

Supplementary Material

## Data Availability

The data that support this study are available from the corresponding authors upon reasonable request. Sequence data from brain organoids bulk RNA‐seq that support the findings of this study have been deposited in the European Nucleotide Archive with the primary accession code PRJEB43363.
