# Supplementary material for: Inhibition of the cGAS‐STING pathway ameliorates the premature senescence hallmarks of Ataxia‐Telangiectasia brain organoids
Source: Aging Cell. 2021 Aug 30;20(9):e13468. doi: 10.1111/acel.13468 (PMC8441292; doi:10.1111/acel.13468)
Supplement: Supplementary file 1 — Supplementary Material [file ACEL-20-e13468-s001.pdf]

Supplementary Figure 1

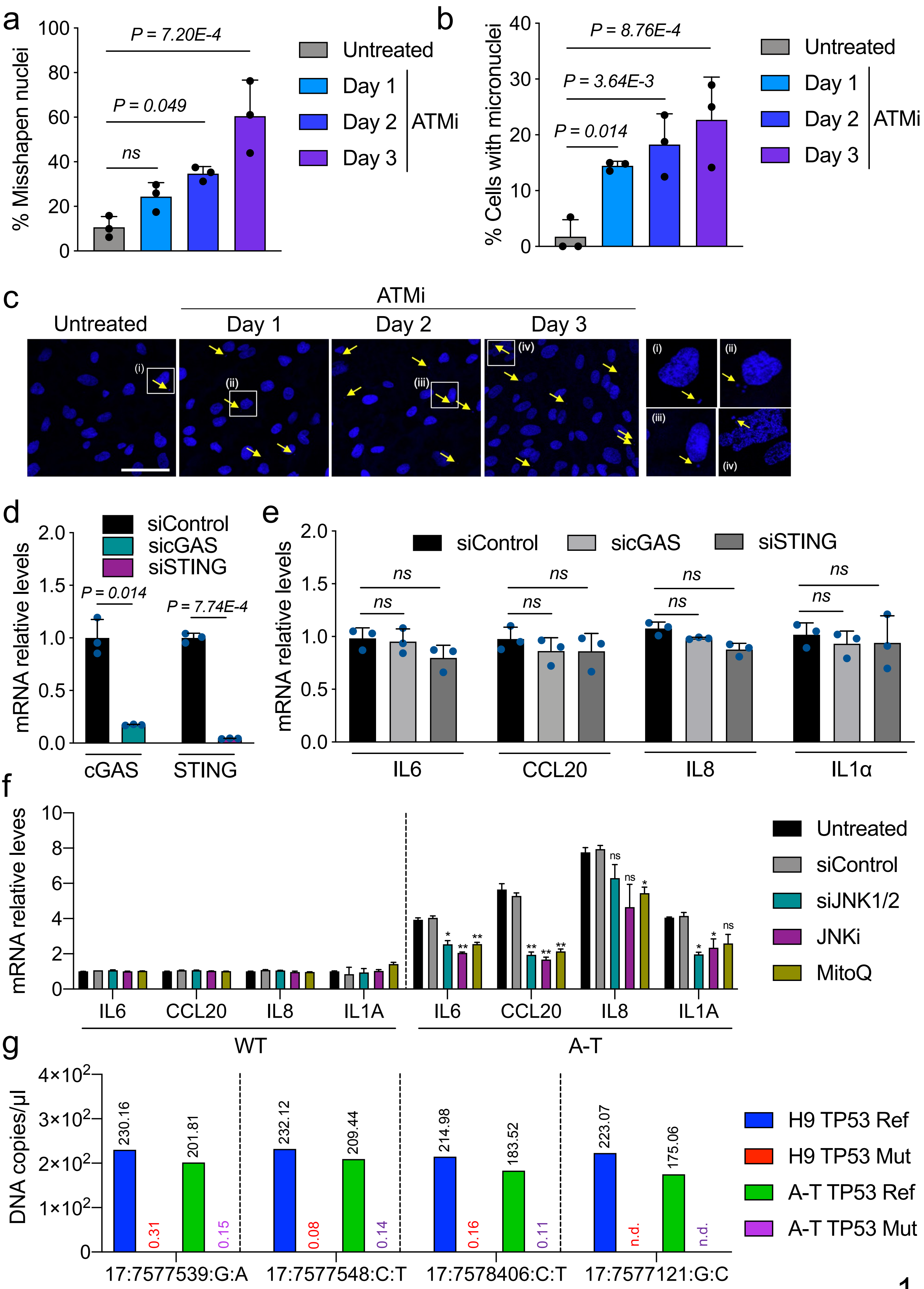

Supplementary Figure 1. (a-c) Human dermal fibroblasts were treated with 10 µM ATM inhibitors (ATMi) for up to three days and monitored over time to assess **(a)** nuclear shape and **(b)** percentage of micronuclei. n=3 independent experiments; one-way ANOVA with Tukey's multiple-comparison post-hoc corrections. **(c)** Representative images from quantifications shown in **a** and **b**. Arrowheads indicate micronuclei. Scale bar, 50 µm. **(d,e)** ONS cells were transfected with the indicated siRNA for 48hr. **(d)** Total RNA from A-T ONS cells in Figs. 1d, e was used for RT-qPCR to detect cGAS and STING mRNA levels and normalized to RPLP0 mRNA and compared to siControl. Error bars represent s.d.; n=3 independent experiments. **(e)** Total RNA from WT ONS cells was used for RT-qPCR to detect the indicated SASP genes and normalized to RPLP0 mRNA and compared to siControl. Error bars represent s.d.; ns: not significant.; n=3 independent experiments; one-way ANOVA with Tukey's multiple-comparison post-hoc corrections. **(f)** WT and A-T ONS cells were either transfected with the indicated siRNAs, treated with JNK inhibitor (JNKi, SP600125, 20 µM) or MitoQ (100 nM) for ten days. Total RNA from these cells was thereafter used for RT-qPCR to detect the indicated SASP genes and normalized to RPLP0 mRNA and compared to untreated controls. Error bars represent s.d.; ns: not significant.; n=2 independent experiments; one-way ANOVA with Tukey's multiple-comparison post-hoc corrections. **(g)** Genomic DNA from H9 and A-T pluripotent stem cells was used to perform ddPCR for the identification of TP53 mutations. The number of droplets positive is indicated at the top of each bar (in blue and green = droplets containing wild-type TP53 DNA sequence, in red and magenta = droplets containing mutated TP53 DNA sequence). 17:7577539:G:A, 17:7577548:C:T, 17:7578406:C:T and 17:7577121:G:C represent the TP53 genomic regions spanned by primers shown in Table S2 and named p53 #1, 2 3 and 4, respectively. n.d.: not detected.

Supplementary Figure 2

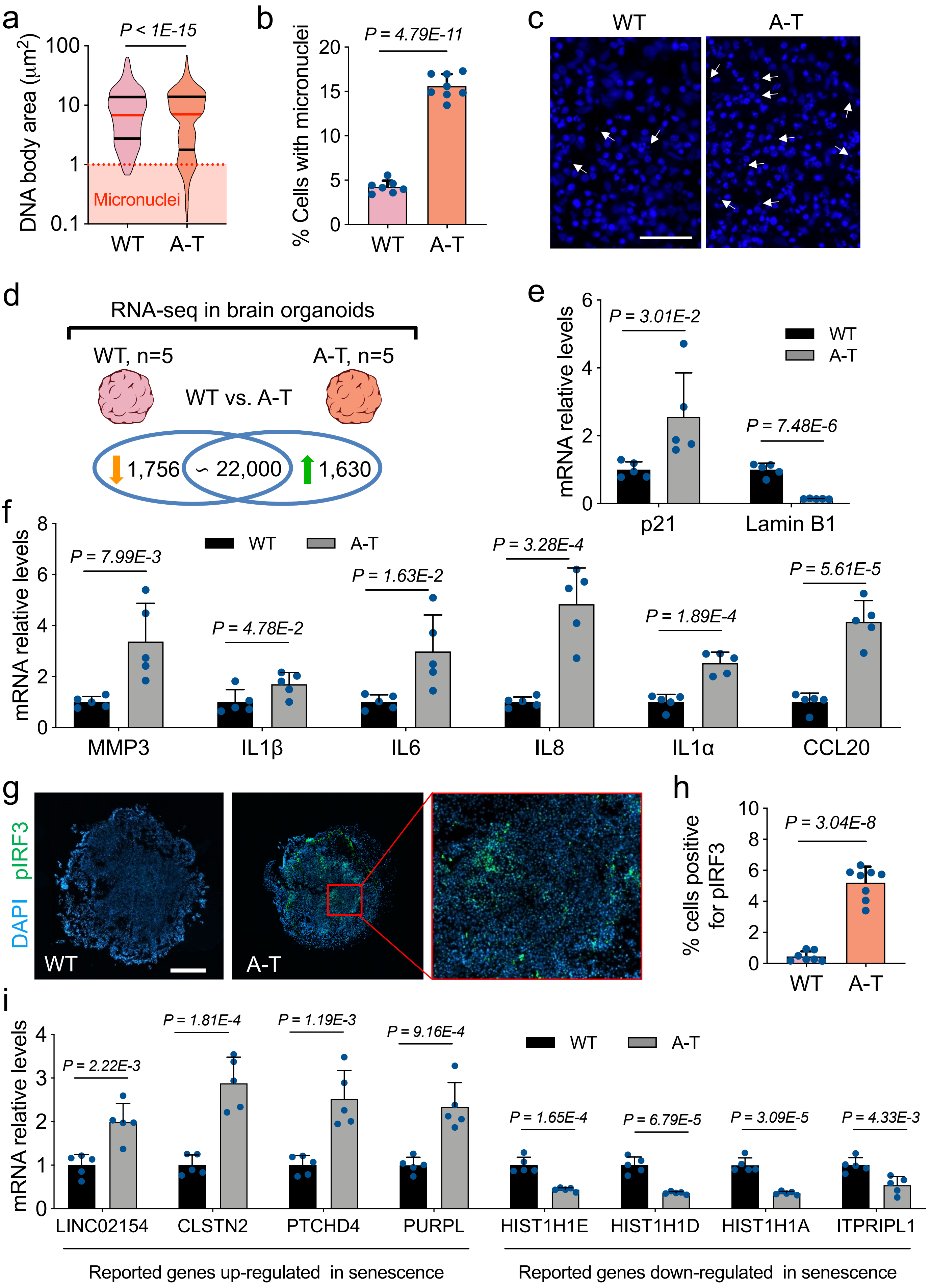

Supplementary Figure 2. (a-i) WT and A-T Brain organoids (BOs) were generated and grown *in vitro* for 3 months and collected for analysis. **(a)** BO sections stained with DAPI were quantified and DNA object distributions were displayed in the form of violin plots. Objects or DNA body areas below 1 μm^2 were identified as micronuclei. At least 20,000 objects were counted, n=3 independent experiments; median and quartiles are depicted in red and black lines respectively; ***P < 0.001; Student's t-test. **(b)** The fraction of micronuclei identified in **a** were quantified over the total amount of cells. n=3 independent experiments; error bars represent s.d.; ***P < 0.001; Student's t-test. **(c)** Representative images from quantifications shown in **a** and **b**. Arrowheads indicate micronuclei. Scale bar, 100 μm. **(d)** Venn diagram shows differentially expressed genes in BOs (WT, n = 5; A-T n = 5 independent biological samples) defined with a significance adjusted P value <0.05. **(e,f)** Total RNA from human A-T BOs was used to quantify the mRNA expression levels of the indicated genes and normalized to B2M mRNA and compared to WT BO controls. n=5 independent biological samples; error bars represent s.d.; Student's t-test. **(g)** Immunofluorescence representative images of WT and A-T BO sections stained for IRF3 phosphorylated on serine 386 (pIRF3). Scale bar, 0.7 mm. **(h)** Quantification of data presented in **g**. Bar graphs show the percentage of pIRF3 positive cells. Each point in the scatter plot represents a single BO section analysed. Error bars represent s.d.; n=3 independent experiments; Student's t-test. **(i)** Total RNA from human A-T BOs was used to quantify the mRNA expression levels of the indicated senescence-associated genes and normalized to B2M mRNA and compared to WT BO controls. n=5 independent biological samples; error bars represent s.d.; Student's t-test.

Supplementary Figure 3

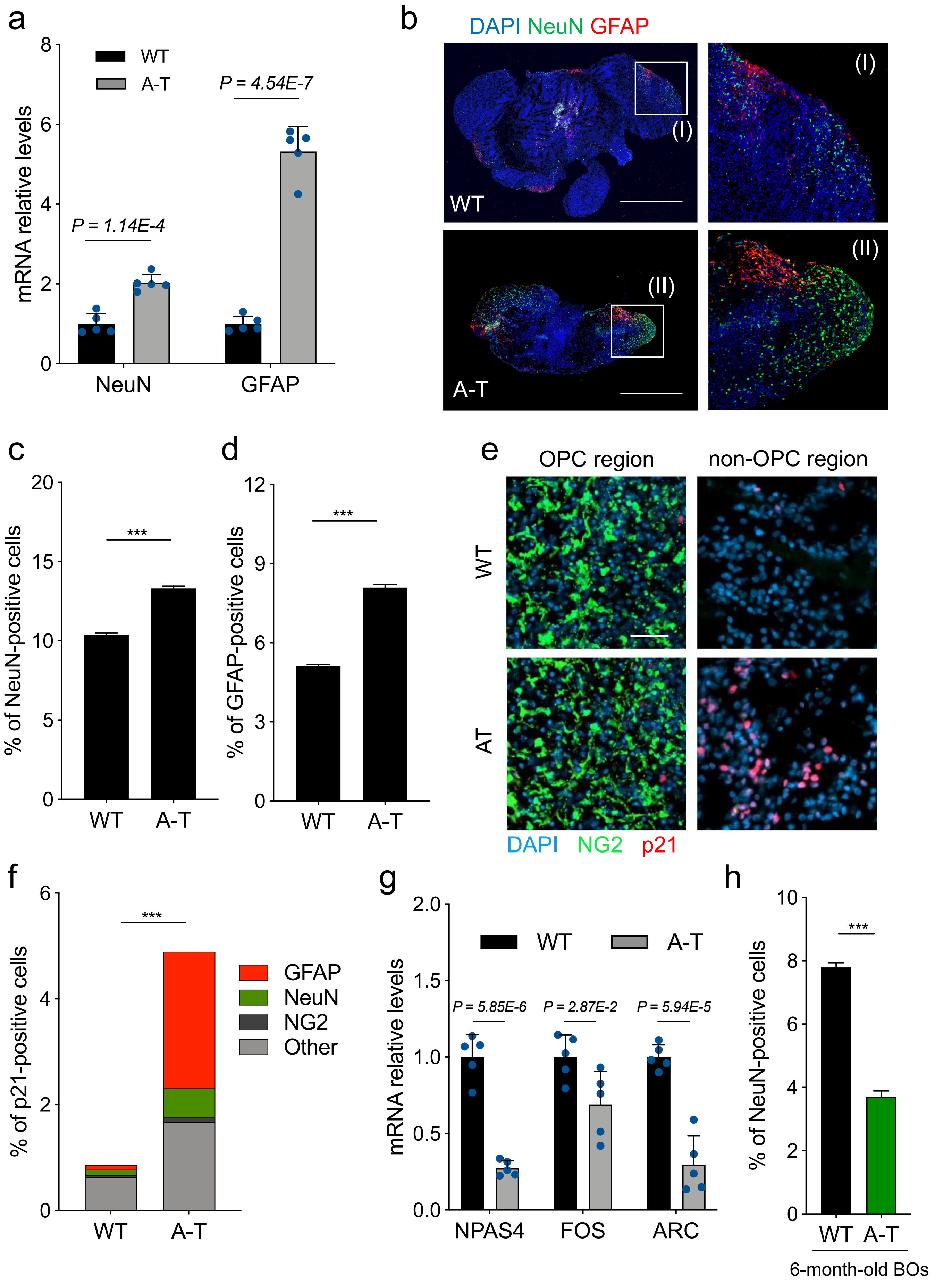

Supplementary Figure 3. (a-f) WT and A-T Brain organoids (BOs) were generated and grown *in vitro* for 3 months and collected for analysis. **(a)** Total RNA from human A-T BOs was used to quantify the mRNA expression levels of NeuN and GFAP; and normalized to B2M mRNA and compared to WT BO controls. n=5 independent biological samples; error bars represent s.d.; Student's t-test. **(b-f)** BO sections were stained for the indicated markers. **(b)** Immunofluorescence representative images of WT and A-T BO sections stained for NeuN (green) and GFAP (red). Scale bar, 0.7 mm. **(c,d)** Quantification of data presented in **b**. Bar graphs show the percentage of NeuN and GFAP positive cells ± 95% confidence interval. n = 3 independent biological samples; at least 100,000 cells per sample have been analysed; ***P < 0.001, chi-squared test. **(e)** Immunofluorescence representative images of WT and A-T BO sections stained for NG2 (green) and p21 (red). Scale bar, 50 μm. Oligodendrocyte progenitor cell (OPC) regions are those BO section areas positive for the OPC marker NG2 and non-OPC regions are those BO areas where NG2 was not detected. **(f)** BO sections stained for NeuN, GFAP, NG2 and p21 were quantified. At least 100,000 cells per condition were analysed. Error bars represent s.d.; n = 3 independent biological samples. ***P < 0.001; Student's t-test. **(g)** Total RNA from human A-T BOs was used to quantify the mRNA expression levels of the indicated Immediate Early Genes and normalized to B2M mRNA and compared to WT BO controls. n=5 independent biological samples; error bars represent s.d.; Student's t-test. **(h)** Quantifications of NeuN positive cells in 6-month-old WT and A-T BOs. Error bars represent 95% confidence interval. n = 3 independent biological samples; at least 40,000 cells per sample have been analysed; ***P < 0.001, chi-squared test.

Supplementary Figure 4

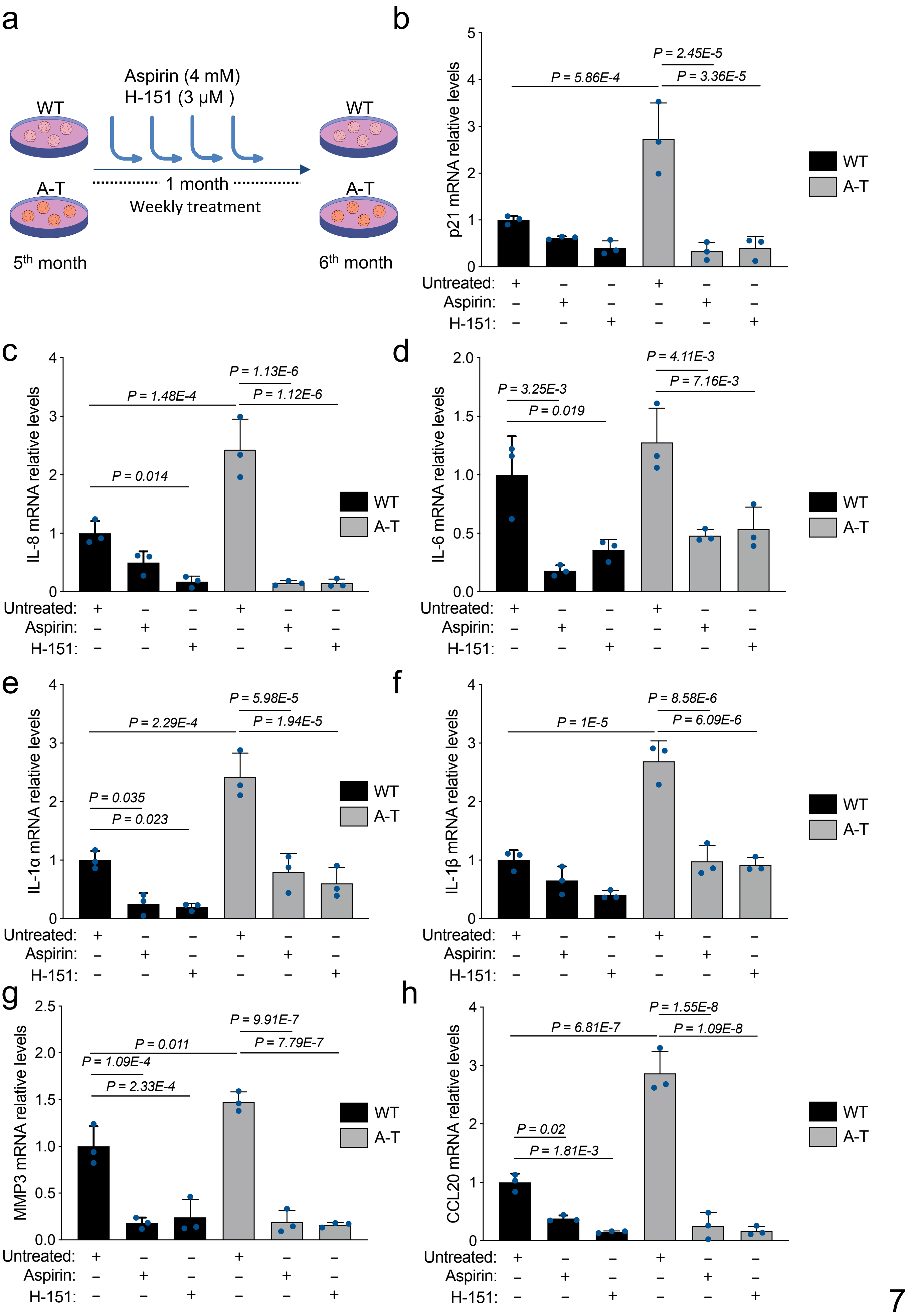

Supplementary Figure 4. (a) Schematic illustration of the experimental design of aspirin and H-151 treatments in WT and A-T BOs. Briefly, 5-month-old BOs were treated once a week with either aspirin (4 mM) or H-151 (3 μM) for 1 month. After the four rounds of treatments, BOs were collected, analysed and compared with the untreated 6-month-old counterparts. **(b-h)** Total RNA from human WT and A-T BOs treated as described in panel **a** was used to quantify the mRNA expression levels of the indicated genes and normalized to B2M mRNA and compared to untreated WT BO controls. n=3 independent biological samples; error bars represent s.d.; one-way ANOVA with Tukey's multiple-comparison post-hoc corrections.

Supplementary Figure 5

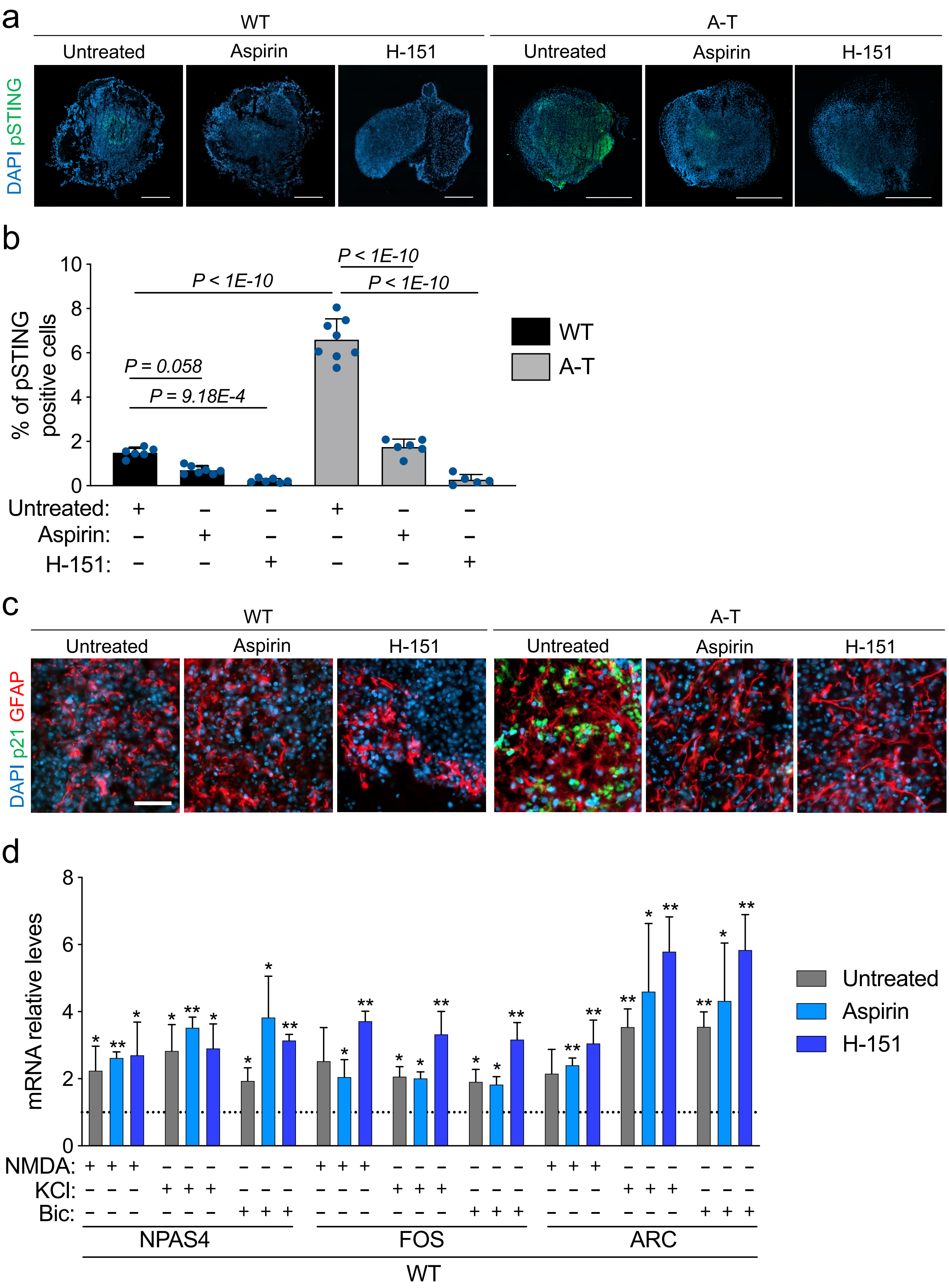

Supplementary Figure 5. (a-c) WT and A-T BOs were treated for one month with aspirin (4mM) or H-151 (3 µM) starting at 5 months of development. **(a)** Immunofluorescence representative images of BO sections stained for STING phosphorylated on serine 366 (pSTING). Scale bar, 0.7 mm. **(b)** Quantification of data presented in **a**. Bar graphs show the percentage of pSTING positive cells. Each point in the scatter plot represents a single BO section analysed. Error bars represent s.d.; n=3 independent experiments; one-way ANOVA with Tukey's multiple-comparison post-hoc corrections. **(c)** Immunofluorescence representative images of BO sections stained for p21 (green) and GFAP (red) from quantifications shown in Figure 5b. Scale bar, 50 µm. **(d)** WT BOs at 5 months of development exposed for 4 weeks to either aspirin (4mM) or H-151 (3 µM) were treated with NMDA (100 µM), KCl (55mM) and Bicuculline (50 µM) for 30 minutes. Immediately after, BOs were collected for RNA extraction. RT-qPCR analysis of the indicated IEGs was performed, and B2M mRNA was used as normalizer. Bar graphs show fold induction of IEG levels in stimulated organoids relative to untreated (no neuronal-stimulating drug, depicted by a grid line). Error bars represent s.d.; n=3 independent experiments. *P<0.05, **P<0.01, Each bar was compared to grid line values; one-way ANOVA with Tukey's multiple-comparison post-hoc corrections.

Supplementary Table 1

Primer		Sequence (5'-3' orientation)	Primer		Sequence (5'-3' orientation)
B2M	Fw	TTCTGGCCTGGAGGCTATC	CDKN1A	Fw	TCACTGTCTTGTACCCTTGTGC
	Rv	TCAGGAAATTTGACTTTCCATTC		Rv	GGCGTTTGGAGTGGTAGAAAT
RPLP0	Fw	TTCATTGTGGGAGCAGAC	LINC02154	Fw	ACTGCGCCACCTCTGATATG
	Rv	CAGCAGTTTCTCCAGAGC		Rv	GACCCACTGATTGTGCCTGA
IL8	Fw	TTGGCAGCCTTCCTGATTTC	CLSTN2	Fw	CCTCAAAGTATCCTCCAAAGTCC
	Rv	TCTTTAGCACTCCTTGGCAAAAC		Rv	CATAGGCATCTACCTCAGGGATAC
IL1A	Fw	GGTTGAGTTTAAGCCAATCCA	PTCHD4	Fw	TCTTCATCACCGATGGAAAGT
	Rv	TGCTGACCTAGGCTTGATGA		Rv	CATCCGGACAGAAGCTCAA
IL6	Fw	CAGCCCTGAGAAAGGAGACAT	PURPL	Fw	CCGGCTATTTTGGAGATTGA
	Rv	GGTTCAGGTTGTTTTCTGCCA		Rv	AGCCTGGACCACAGAACAAG
CCL20	Fw	AACCATGTGCTGTACCAAGAGT	HIST1H1E	Fw	CTAAACCAAAGACCGCCAAG
	Rv	AAGTTGCTTGCTTCTGATTCGC		Rv	GGGTTGTGTTGGGCTTCTAA
IL1B	Fw	CTCTCTCCTTTCAGGGCCAA	HIST1H1D	Fw	AAGGCAGTGGCAGCTTCTAA
	Rv	GAGAGGCCTGGCTCAACAAA		Rv	CCAAGCTTGATACGGCTGTT
MMP3	Fw	GGATGCCAGGAAAGGTTCTG	HIST1H1A	Fw	AGGCTACGACGTGGAGAAGA
	Rv	CCAGGTGTGGAGTTCCTGATGT		Rv	GAGGACGCCTTCTTGTTGAG
STING	Fw	ATATCTGCGGCTGATCCTGC	ITPRIPL1	Fw	CCAAGTTCCAAAGGGAGGAT
	Rv	TTGTAAGTTCGAATCCGGGC		Rv	GCCAAGAACAGCAGGCTTAT
CGAS	Fw	GGCGGTTTTGGAGAAGTTGA	LMNB1	Fw	CTCTCGTCGCATGCTGACAG
	Rv	GCCGCCGTGGAGATATCAT		Rv	TCCCTTATTTCCGCCATCTCT
FOS	Fw	TGCAGCCAAATGCCGCAAC	RBFOX3	Fw	CAAGCGGCTACACGTCTCCAACAT
	Rv	TCGGTGAGCTGCCAGGATG		Rv	GCTCGGTCAGCATCTGAGCTAGT
NPAS4	Fw	GTGAGGCTACAGGCCAAGAC	GFAP	Fw	CCGACAGCAGGTCCATGTG
	Rv	AGGGCAGCATGGTCGGAGTG		Rv	GTTGCTGGACGCCATTGC
ARC	Fw	AAGTCGCACACGCAGCAGAGCA			
	Rv	AGGCGGGCGTGAATCACTGGA			

Fw: Forward; Rv: Reverse

Supplementary Table 2

Primer		Sequence (5’-3’ orientation)	Probe	Sequence (5’-3’ orientation)
p53 #1	Fw	TGGAGTCTTCCAGTGTGATG	p53 #1 Ref	HEX-ATGGGCCTCCGGTTCATGC-IBQ
	Rv	TGTAACAGTTCCTGCATGGG	p53 #1 Mut	FAM-ATGGGCCTCCAGTTCATGCC-IBQ
p53 #2	Fw	ACATGTGTAACAGTTCCTGC	p53 #2 Ref	HEX-TGGGCGGCATGAACCGGA-IBQ
	Rv	TGGAGTCTTCCAGTGTGATG	p53 #2 Mut	FAM-ATGGGCAGCATGAACCGGA-IBQ
p53 #3	Fw	TCTACAAGCAGTCACAGCAC	p53 #3 Ref	HEX-TGAGGCGCTGCCCCCACC-IBQ
	Rv	CTCACCATCGCTATCTGAGC	p53 #3 Mut	FAM-TGAGGCACTGCCCCCACC-IBQ
p53 #4	Fw	TCTCTTCCTCTGTGCGCC	p53 #4 Ref	FAM-ACAAACACGCACCTCAAAGCTG-IBQ
	Rv	TGGTAATCTACTGGGACGGAA	p53 #4 Mut	HEX-CACAAACACCCACCTCAAAGCTG-IBQ

Fw: Forward; Rv: Reverse. For probes, a 5' Reporter Dye (FAM or HEX) and a 3′ quencher (Iowa Black with Zen, IBQ) were added to the oligonucleotide backbone.
